# Multi-Omics Analysis of Morbid Obesity Using a Patented Unsupervised Machine Learning Platform: Genomic, Biochemical, and Glycan Insights

**DOI:** 10.3390/ijms27031551

**Published:** 2026-02-04

**Authors:** Irena Šnajdar, Luka Bulić, Andrea Skelin, Leo Mršić, Mateo Sokač, Maja Brkljačić, Martina Matovinović, Martina Linarić, Jelena Kovačić, Petar Brlek, Gordan Lauc, Martina Smolić, Dragan Primorac

**Affiliations:** 1 St. Catherine Specialty Hospital, 10000 Zagreb, Croatialuka.bulic@svkatarina.hr (L.B.);; 2 Department of Surgery, University Hospital Center Zagreb, 10000 Zagreb, Croatia; 3 School of Medicine, Josip Juraj Strossmayer University of Osijek, 31000 Osijek, Croatia; 4 Genos Glycoscience Research Laboratory, 10000 Zagreb, Croatia; 5 Algebra Bernays University, 10000 Zagreb, Croatia; 6 Department of Molecular Biology, Faculty of Science, University of Zagreb, 10000 Zagreb, Croatia; 7 Faculty of Pharmacy and Biochemistry, University of Zagreb, 10000 Zagreb, Croatia; 8 Faculty of Dental Medicine and Health, Josip Juraj Strossmayer University of Osijek, 31000 Osijek, Croatia; 9 Forensic Science Program, Department of Biochemistry & Molecular Biology, The Pennsylvania State University, State College, PA 16802, USA; 10 Henry C. Lee College of Criminal Justice and Forensic Sciences, University of New Haven, West Haven, CT 06516, USA; 11 Sana Kliniken Oberfranken, 96450 Coburg, Germany; 12 School of Medicine, University of Split, 21000 Split, Croatia; 13 Medical School, University of Rijeka, 51000 Rijeka, Croatia; 14 Medical School, University of Mostar, 88000 Mostar, Bosnia and Herzegovina; 15 National Forensic Sciences University, Gandhinagar 382007, India; 16 School of Medicine, University of Pittsburgh, Pittsburgh, PA 15213, USA

**Keywords:** morbid obesity, whole-genome sequencing, glycosylation, machine learning, personalized medicine

## Abstract

Morbid obesity is a complex, multifactorial disorder characterized by metabolic and inflammatory dysregulation. The aim of this study was to observe changes in obese patients adhering to a personalized nutrition plan based on multi-omic data. This study included 14 adult patients with a body mass index (BMI) > 40 kg/m^2^ who were consecutively recruited from those presenting to our outpatient clinic and who met the inclusion criteria. Clinical, biochemical, hormonal, and glycomic parameters were assessed, along with whole-genome sequencing (WGS) that included a focused analysis of obesity-associated genes and an extended analysis encompassing genes related to cardiometabolic disorders, hereditary cancer risk, and nutrigenetic profiles. Patients were stratified into nutrigenetic clusters using a patented unsupervised machine learning platform (German Patent Office, No. DE 20 2025 101 197 U1), which was employed to generate personalized nutrigenetic dietary recommendations for patients with morbid obesity to follow over a six-month period. At baseline, participants exhibited elevated glucose, insulin, homeostatic model assessment for insulin resistance (HOMA-IR), triglycerides, and C-reactive protein (CRP) levels, consistent with insulin resistance and chronic low-grade inflammation. The majority of participants harbored risk alleles within the fat mass and obesity-associated gene (*FTO*) and the interleukin-6 gene (*IL-6*), together with multiple additional significant variants identified across more than 40 genes implicated in metabolic regulation and nutritional status. Using an AI-driven clustering model, these genetic polymorphisms delineated a uniform cluster of patients with morbid obesity. The mean GlycanAge index (56 ± 12.45 years) substantially exceeded chronological age (32 ± 9.62 years), indicating accelerated biological aging. Following a six-month personalized nutrigenetic dietary intervention, significant reductions were observed in both BMI (from 52.09 ± 7.41 to 34.6 ± 9.06 kg/m^2^, *p* < 0.01) and GlycanAge index (from 56 ± 12.45 to 48 ± 14.83 years, *p* < 0.01). Morbid obesity is characterized by a pro-inflammatory and metabolically adverse molecular signature reflected in accelerated glycomic aging. Personalized nutrigenetic dietary interventions, derived from AI-driven analysis of whole-genome sequencing (WGS) data, effectively reduced both BMI and biological age markers, supporting integrative multi-omics and machine learning approaches as promising tools in precision-based obesity management.

## 1. Introduction

Obesity is a chronic, multifactorial metabolic disorder that has been recognized by the World Health Organization as one of the most significant global public health challenges among adults. Current epidemiological evidence suggests that nearly half (45%) of adults worldwide are either overweight or obese, underscoring the growing global burden of obesity. Projections suggest that by 2050, almost 2 in 3 adults over the age of 25 years will have a body mass index (BMI) exceeding 25 kg/m^2^ [1].

BMI remains the primary anthropometric parameter used in the clinical definition of obesity. According to established clinical guidelines, a BMI of 18.5–24.9 kg/m^2^ is considered normal. Overweight status is defined as a BMI between 25.0 and 29.9 kg/m^2^, whereas obesity is diagnosed when BMI exceeds 30 kg/m^2^. Furthermore, obesity is stratified into three classes:

- Class I obesity: BMI 30.0–34.9 kg/m^2^;
- Class II obesity: BMI 35.0–39.9 kg/m^2^;
- Class III obesity (morbid or severe obesity): BMI ≥ 40.0 kg/m^2^ [2].

Obesity is a complex condition influenced by both genetic and environmental factors. The first epidemiological investigation into the hereditary basis of obesity dates back approximately a century. Subsequent studies have consistently demonstrated that genetic heritability accounts for approximately 40–50% of obesity risk in the general population, with heritability estimates exceeding 80% in individuals with morbid (Class III) obesity [3].

Obesity is now recognized as a chronic low-grade inflammatory state that precipitates a cascade of metabolic disturbances, including insulin resistance, type 2 diabetes mellitus, dyslipidemia, hypertension, cardiovascular disease, and malignancies [4]. Adipose tissue, beyond serving as an energy reservoir, functions as an active endocrine organ. Adipocytes secrete leptin, a hormone with pro-inflammatory properties. In the context of hypertrophic adipocytes, typically observed in obesity, there is increased secretion of pro-inflammatory cytokines such as tumor necrosis factor-alpha (TNF-α) and interleukin-6 (IL-6). These cytokines sustain local inflammation within adipose tissue and promote adipocyte apoptosis. The resulting recruitment and activation of macrophages amplify the inflammatory response, further increasing systemic inflammation and contributing to the development of metabolic syndrome and tumorigenesis [5]. Given the complexity of these interrelated inflammatory and metabolic processes, recent multi-omics approaches have provided valuable insights into the molecular mechanisms underlying inflammation, particularly highlighting the role of glycosylation. Glycosylation, as one of the most essential and complex post-translational modifications, critically regulates protein function and modulates a wide spectrum of physiological and pathological pathways. Alterations in N-glycosylation of immunoglobulin G (IgG) have been linked to aging and various chronic inflammatory disorders [6,7,8,9]. Emerging evidence suggests a potential pro-inflammatory role for specific IgG glycan profiles in individuals with obesity, although further validation is required [10].

In the past decade and a half, the integration of high-throughput genotyping technologies with comprehensive analytical approaches such as genome-wide association studies (GWAS) has substantially advanced the understanding of the genetic architecture of obesity, leading to the identification of more than 500 susceptibility genes [11,12]. Genetically determined obesity may be categorized as syndromic or non-syndromic. Syndromic obesity results from chromosomal aberrations and is typically accompanied by additional somatic and neurodevelopmental anomalies. Notable examples include Prader–Willi syndrome (paternal deletion of 15q), Fragile X syndrome, WAGR syndrome, and Cohen syndrome [13].

Non-syndromic forms of obesity may arise from monogenic or polygenic mechanisms. Monogenic forms, although rare, often involve mutations in genes related to leptin signaling, such as *FTO*, *LEP*, *MC4R*, *LEPR*, and *POMC*, leading to profound disturbances in appetite regulation. Analyses of the protein-coding portion of the genome (constituting approximately 1.5–3% of the total genome) have shown that polygenic inheritance accounts for approximately 60% of genetically mediated obesity. Although over 500 genes have been implicated, their penetrance and effect sizes vary significantly [3,4]. Advances in whole-genome sequencing (WGS) technologies, further accelerated by the application of artificial intelligence (AI), now enable a comprehensive elucidation of the genetic architecture of obesity and support the development of predictive screening strategies for at-risk populations [14,15].

The rapid advancement of artificial intelligence (AI) technologies has transformed both scientific research and clinical practice. Machine learning (ML) employs statistical algorithms to construct predictive models capable of learning from large datasets and generalizing to novel data inputs [16,17,18]. Among ML approaches, unsupervised learning algorithms, particularly clustering techniques, are instrumental in exploratory data analysis. These algorithms identify latent patterns within high-dimensional, unlabeled datasets using complex, multi-parametric models [19].

While genetic susceptibility underlies obesity risk, diet remains a crucial modifiable factor influencing metabolic and inflammatory pathways. Nutrition is therefore increasingly recognized as a therapeutic strategy capable of modifying energy balance, insulin sensitivity, lipid metabolism, and systemic inflammation. Responses to dietary interventions vary substantially between individuals, reflecting genetic heterogeneity in appetite regulation, nutrient metabolism, and inflammatory signaling. This inter-individual variability forms the basis of nutrigenetics and supports the need for personalized dietary approaches, particularly in severe obesity. Several obesity-associated genes, including *FTO*, *MC4R*, *LEPR*, and inflammatory mediators such as IL-6, have been shown to interact with dietary factors. Diet may also influence immunometabolic processes through modulation of IgG N-glycosylation, which reflects systemic inflammation and biological aging [20,21,22].

This study aimed to integrate genomic, biochemical, and glycomic data to elucidate the molecular mechanisms underlying morbid obesity and to evaluate the effects of personalized nutrigenetic interventions derived from AI-driven analysis of WGS data.

## 2. Results

Baseline clinical and biochemical characteristics are summarized in Table 1 and Table 2.

Most biochemical parameters, including erythrocyte count, hemoglobin, hematocrit, platelets, and leukocytes, were within their respective reference ranges in all participants. However, several metabolic and inflammatory markers deviated from normal values. Fasting glucose levels exceeded the upper reference limit (5.5 mmol/L) in 9 of 14 participants (64%), while HbA1c values indicated impaired glucose metabolism (>5.6%) in 6 participants (43%). Markers of insulin resistance were frequently abnormal: HOMA-IR exceeded the reference threshold (<2) in 11 participants (79%), and fasting insulin levels were above normal (>152.9 mU/L) in 5 participants (36%). Regarding lipid metabolism, triglyceride concentrations were elevated (>1.7 mmol/L) in 6 participants (43%), and HDL cholesterol was below the recommended level (>1.2 mmol/L) in 10 participants (71%). Total cholesterol exceeded the reference value (<5.0 mmol/L) in 8 participants (57%), while LDL cholesterol values were above the optimal range (<3.0 mmol/L) in 12 participants (86%). Vitamin D deficiency (<75 nmol/L) was present in all participants (100%), with a mean concentration of 47.36 ± 13.32 nmol/L. Creatine kinase (CK) activity was elevated (>153 U/L) in 8 participants (57%), and uric acid levels were increased (>337 μmol/L) in 9 participants (64%). Inflammatory parameters were also abnormal: C-reactive protein (CRP) was elevated (>5 mg/L) in 9 participants (64%), and erythrocyte sedimentation rate (ESR) was above normal (>24 mm/3.6 ks) in 5 participants (36%), indicating the presence of chronic low-grade inflammation characteristic of morbid obesity.

**Figure 1:**
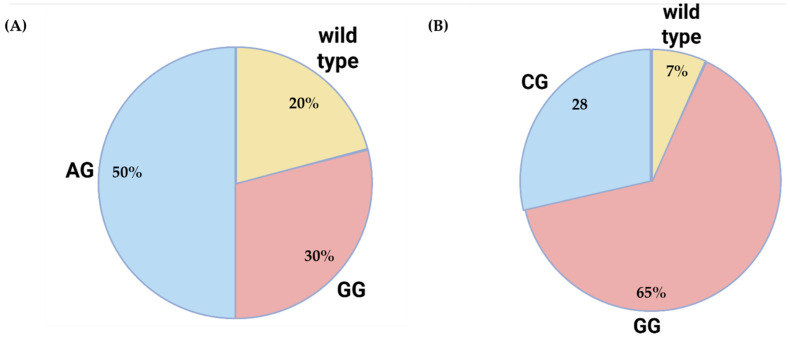
Distribution of obesity- and inflammation-associated gene polymorphisms among study participants: (**A**) Distribution of the FTO rs9939609 genotypes in the study cohort (n = 14). The AG genotype was present in 50% of participants (7/14), the GG genotype in 30% (4/14), and the wild-type (AA) genotype in 20% (3/14). The G allele was associated with increased body mass index (BMI) and a higher risk of obesity. (**B**) Distribution of the IL6 rs1800795 genotypes among the same participants. The GG genotype was identified in approximately 65% of participants (9/14), the CG genotype in 28% (4/14), and the wild-type (CC) genotype in 7% (1/14). Carriers of the G allele may exhibit a higher-than-normal inflammatory response due to increased interleukin-6 (IL-6) expression.

Genetic analysis revealed the presence of multiple gene variants previously associated with elevated BMI and increased risk for obesity in all participants. The most frequently observed variants were within the *FTO* gene, which has been consistently implicated in adiposity-related phenotypes in large-scale genome-wide association studies (GWAS) (Figure 1A).

Although the small sample size limits the power of stratified analyses, preliminary data suggest the enrichment of risk alleles across several cardiometabolic and hereditary cancer-related gene panels. Of particular interest is that 13 out of 14 participants (92.86%) were carriers of the G allele of the *IL-6* gene (interleukin-6), a variant previously linked to increased low-grade chronic inflammation (Figure 1B). This finding suggests a potential genetic predisposition toward a heightened inflammatory profile in this population, consistent with elevated levels of inflammatory biomarkers such as C-reactive protein (CRP) and erythrocyte sedimentation rate (ESR).

**Figure 2:**
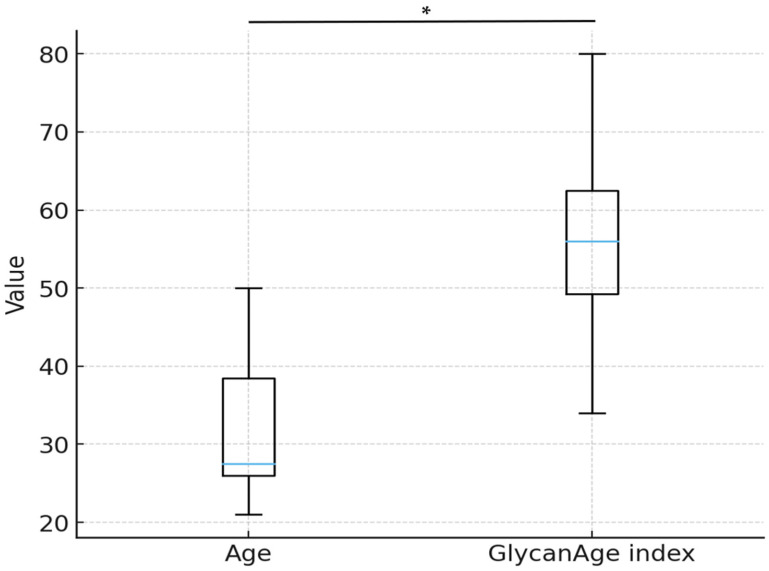
Comparison between chronological age and GlycanAge index in study participants (Wilcoxon signed-rank test, * *p* < 0.01).

Moreover, analysis of the IgG glycome revealed a significantly elevated GlycanAge index, a biomarker of biological age derived from IgG glycosylation patterns. This finding is consistent with prior studies linking altered N-glycosylation to metabolic dysfunction and chronic low-grade inflammation (Table 3, Figure 2).

**Figure 3:**
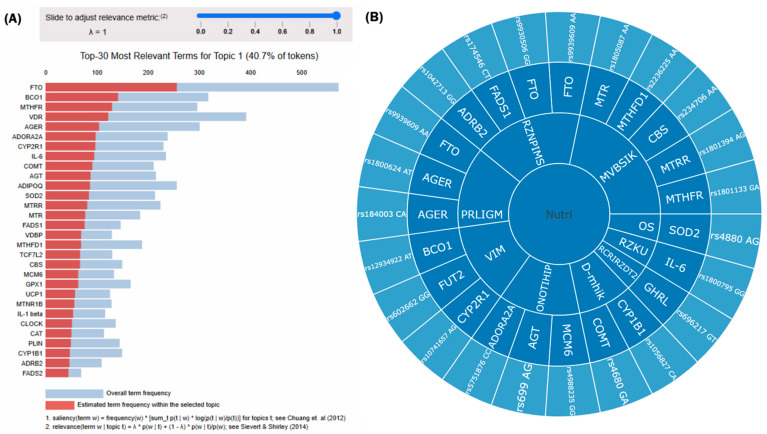
Example of a nutrigenetic profile of a patient classified into Cluster 1 based on AI-driven analysis of whole-genome sequencing (WGS) data: (**A**) The top 30 genes with the highest relevance scores within Cluster 1, representing 40.7% of all identified genetic variants. The red bars indicate the estimated term (gene) frequency within the selected topic, whereas blue bars represent the overall term frequency across all topics. (**B**) A visual representation of Cluster 1 characteristics. The inner ring represents nutrition-based gene groups, the middle ring represents genes, and the outer ring represents respective polymorphisms. RZNPIMS—risk of obesity and metabolic syndrome, MVBSIK—altered vitamin B group and choline metabolism, OS—oxidative stress, RZKU—chronic inflammation, RCRIRZDT2—circadian rhythm regulation and diabetes type 2 risk, D-mhik—detoxication (hormone and xenobiotic metabolism), PRLGM—dysregulation of lipid and glucose metabolism, VIM—related to vitamins and minerals.

Using the patented digital platform for nutrigenetic analysis, study participants were categorized based on specific genetic findings. All 14 participants were classified into Cluster 1 (Figure 3).

**Figure 4:**
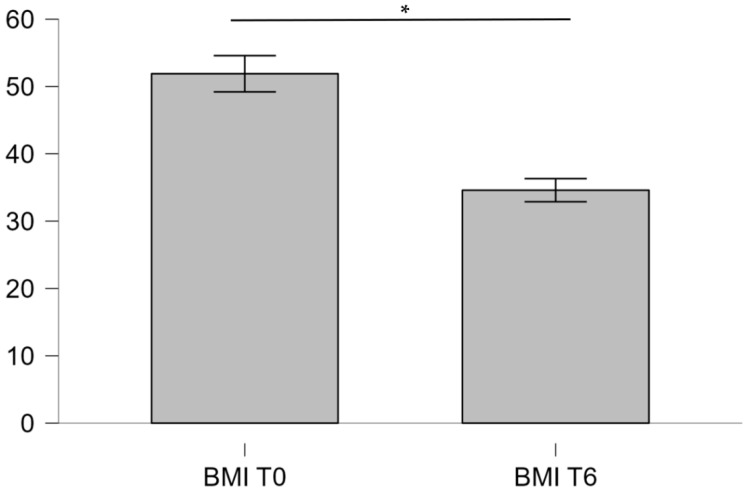
Comparison of initial BMI and BMI after 6 months (Wilcoxon signed-rank test, * *p* < 0.01).

**Figure 5:**
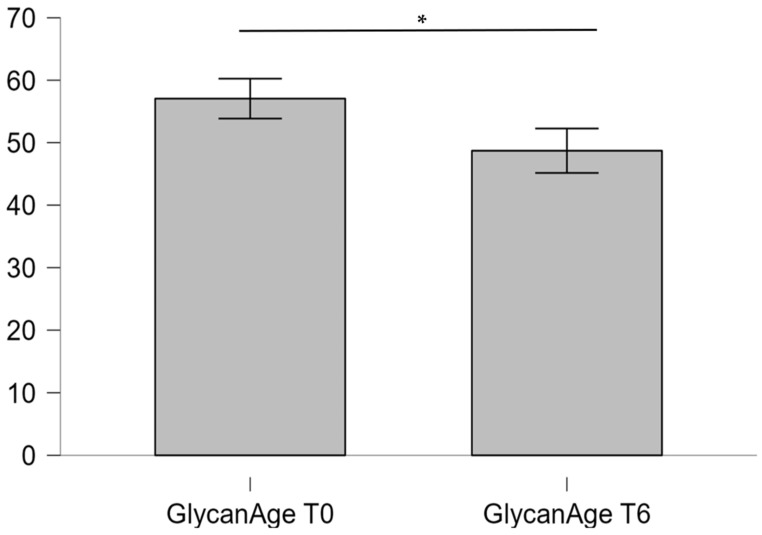
Statistical analysis of GlycanAge index before and after 6 months (Wilcoxon signed-rank test, * *p* < 0.01).

After six months of personalized dietary intervention, both BMI and the GlycanAge index demonstrated statistically significant improvement (Table 4 and Table 5, Figure 4 and Figure 5).

## 3. Discussion

This study analyzed biochemical, genetic, and glycomic signatures of individuals with morbid obesity and evaluated the effects of a personalized nutrigenetic intervention over six months. The findings demonstrate that morbid obesity is characterized by a combination of metabolic dysregulation, chronic low-grade inflammation, and accelerated biological aging, reflected by altered immunoglobulin G N-glycosylation patterns. Importantly, the personalized dietary intervention guided by nutrigenetic profiling led to significant improvements in BMI and GlycanAge index, suggesting a partial reversal of metabolic and glycomic abnormalities [23].

The baseline biochemical profile of the cohort was consistent with established features of obesity-related metabolic dysfunction [24]. Elevated fasting glucose, insulin, and HOMA-IR indices pointed to insulin resistance, a major driver of obesity-associated morbidity. Dyslipidemia, particularly elevated triglycerides and reduced HDL cholesterol, further indicated an unfavorable cardiometabolic risk profile. Increased C-reactive protein (CRP) and erythrocyte sedimentation rate (ESR) levels confirmed a chronic, low-grade inflammatory state originating from hypertrophic and dysfunctional adipose tissue. Such inflammatory activation contributes not only to metabolic disturbances but also to endothelial dysfunction and premature vascular aging.

Within Cluster 1, AI-driven analysis identified the top 30 genes most relevant to the cluster’s genetic architecture, encompassing loci involved in metabolic regulation, nutrient metabolism, and inflammatory signaling pathways. The high prevalence of risk variants in the *FTO* and *IL6* genes among participants provides a plausible genetic basis for the observed metabolic dysregulation and chronic low-grade inflammation. The *FTO* variants detected in this cohort, including *rs9939609*, have been associated with altered expression of appetite-regulating pathways, resulting in increased energy intake, reduced satiety, and higher BMI in diverse populations [25]. The high prevalence of the *IL6 rs1800795* G allele (92.86%) suggests a genetic predisposition to elevated *IL-6* synthesis and chronic low-grade inflammation. The concurrent presence of both polymorphisms may potentiate a phenotype prone to energy imbalance and chronic inflammation, potentially accelerating metabolic aging. These findings support the view that genetic predisposition amplifies the adverse metabolic effects of obesity and may modulate individual responses to lifestyle interventions.

The markedly increased GlycanAge index relative to chronological age observed in this cohort reflects the profound systemic impact of obesity on biological aging. IgG glycosylation patterns are tightly regulated by inflammatory and metabolic cues; pro-inflammatory, agalactosylated glycans dominate in obesity and type 2 diabetes, reflecting a state of chronic immune activation. The elevated GlycanAge index thus provides molecular evidence of accelerated biological aging driven by obesity-associated inflammation. The significant reduction in GlycanAge index after six months of intervention suggests that these glycomic signatures are reversible and sensitive to metabolic improvement [26,27].

Following the personalized dietary plan derived from nutrigenetic clustering, participants achieved a substantial reduction in both BMI and GlycanAge index. The mean BMI decreased from 52.09 ± 7.41 to 34.6 ± 9.06 kg/m^2^, while the GlycanAge index dropped from 56 ± 12.45 to 48 ± 14.83 years. The observed benefits can be associated with improved insulin sensitivity and decreased systemic inflammation, in part due to a genetics-based optimization of diet. The integration of genomic profiling with lifestyle intervention thus represents a promising model for precision obesity management [28].

Nevertheless, it is important to note that previous studies have reported a short-term increase in glycan levels in association with higher physical activity, suggesting that the observed decrease in glycosylation is likely not a result of exercise-related effects [29]. Therefore, the reduction in glycan markers observed in this study can be primarily attributed to personalized dietary intervention rather than to physical activity. The consistent improvements across clinical, biochemical, and molecular parameters strongly support the beneficial impact of the individualized nutrigenetic intervention. Future research should aim to validate these findings in larger, controlled cohorts, incorporating long-term follow-up to assess the durability of metabolic and glycomic changes. Functional studies exploring the mechanistic links between specific genetic variants, glycosylation patterns, and inflammatory signaling could further clarify the pathways connecting obesity and biological aging.

The strengths of this study lie in its comprehensive multi-omic framework, integrating whole-genome sequencing, glycomic profiling, and biochemical parameters, as well as in the exploratory application of an unsupervised machine learning-based nutrigenetic clustering approach. Nevertheless, several important limitations must be acknowledged. First, the small sample size (n = 14) substantially limits statistical power, increases uncertainty around effect size estimates, and precludes robust subgroup or multivariable analyses. With n = 14 (paired design), the study is powered to detect only relatively large within-person effects (minimum detectable standardized change approximately *d*_*z*_ ∼ 0.75 at 80% power, α = 0.05), so smaller effects may not be reliably detected. Together with the absence of a control group, this design restricts causal inference, and all observed changes in anthropometric and glycomic outcomes should therefore be interpreted as associative and hypothesis-generating. These findings therefore support the exploratory nature of the study and underscore the need for validation in larger, controlled cohorts.

Secondly, the analytic framework relies on a patented, proprietary machine learning platform. Although the general principles of the unsupervised clustering approach are described, the lack of full algorithmic transparency, external validation, and publicly available reproducibility metrics limits independent verification. Moreover, while the analysis focuses on a single cluster of interest, the characteristics and composition of alternative clusters, as well as explicit criteria used to define cluster homogeneity, are not comprehensively presented, which further constrains interpretability.

Lifestyle adherence and physical activity were self-reported, introducing the possibility of reporting bias and residual confounding. Despite the pronounced weight loss observed, detailed dietary intake data, including energy intake, macronutrient composition, and dietary patterns, were not systematically collected before or after the intervention. This limits the ability to attribute observed metabolic and glycomic changes to specific dietary modifications and to disentangle the effects of dietary composition from other lifestyle factors. Overall, these limitations underscore the exploratory nature of the study and highlight the need for larger, controlled, and externally validated investigations with transparent analytical frameworks to confirm the observed associations and elucidate underlying biological mechanisms.

In summary, this study indicates that morbid obesity may be associated with specific biochemical, genetic, and glycomic alterations. These are indicative of systemic inflammation and accelerated biological aging and are collectively linked to an increased cardiovascular risk. The observed improvements in BMI and GlycanAge index following a personalized, nutrigenetic-guided dietary intervention highlight the potential of integrative, precision-based approaches in obesity management. Incorporating genetic and glycomic biomarkers into routine clinical assessment may enhance the prediction of therapeutic response and facilitate the development of individualized strategies to combat obesity and its metabolic complications.

## 4. Materials and Methods

### 4.1. Patient Population Characteristics and Selection Criteria

This study followed a longitudinal design and was approved by the institutional ethics committee. Participants of both sexes, aged 20 to 55 years, were selected from individuals presenting to our outpatient clinic who met the inclusion criteria. As part of the standard examination protocol during routine annual checkups, participants’ height and weight were measured, and BMI was calculated using the following formula: BMI = weight (kg)/height^2^ (m^2^). Inclusion criteria were morbid obesity, defined as BMI > 40 kg/m^2^, and willingness to lose weight through dietary changes and physical activity. Exclusion criteria were ongoing malignant disease, pregnancy or breastfeeding, severe inflammatory disease (both acute or chronic), current usage of GLP-1 agonist, and a confirmed monogenic cause of obesity by WGS. The case study consisted of 16 individuals with morbid obesity, 2 of whom dropped out before study termination, so data for 14 participants were studied.

### 4.2. Patient Evaluation Procedure

After providing written informed consent, all participants underwent a comprehensive clinical examination and laboratory evaluation, including hematological, biochemical, and hormonal assessments. The following biomarkers were analyzed: complete blood count, creatine kinase (CK), glucose, glycated hemoglobin (HbA1c), total cholesterol, triglycerides, high-density lipoprotein (HDL), low-density lipoprotein (LDL), erythrocyte sedimentation rate (ESR), C-reactive protein (CRP), vitamin D, insulin, and homeostatic model assessment for insulin resistance (HOMA-IR), as well as immunoglobulin G (IgG) glycome profiles.

Venous blood samples (5 mL per tube) were collected via peripheral venipuncture following standard phlebotomy protocols. Biochemical analyses were performed at the certified laboratory of St. Catherine Specialty Hospital using validated and standardized procedures. An aliquot of blood serum (500 µL) from each participant was sent to Genos Ltd. (Zagreb, Croatia) for glycomic analysis. The total plasma IgG glycome was analyzed using hydrophilic interaction ultra-performance liquid chromatography (HILIC-UPLC), whereas the IgG glycome composition was determined by capillary gel electrophoresis with laser-induced fluorescence detection (CGE-LIF).

In addition, all participants underwent whole-genome sequencing (WGS) performed in a genetic laboratory with extensive experience in genomic analysis. The analysis included a focused assessment of obesity-associated genes, with extended interpretation covering 563 cardiometabolic disease–related genes, 43 nutrigenetic genes, and 524 genes associated with hereditary cancer risk.

### 4.3. Whole-Genome Sequencing Methodology

Genomic DNA was isolated from peripheral leukocytes using the Mag-Bind Blood & Tissue DNA HDQ Kit (Omega Bio-Tek, Norcross, GA, USA). DNA quality and quantity were assessed using the Qubit™ 1X dsDNA HS Assay (Life Technologies Corporation, Eugene, OR, USA), NanoDrop spectrophotometry, and the Genomic DNA ScreenTape assay and High-Sensitivity D1000 ScreenTape assay (Agilent Technologies, Waldbronn, Germany). Library preparation was performed using the Illumina DNA Prep (M) Tagmentation Kit (Illumina, Inc., San Diego, CA, USA). The final indexed libraries were sequenced on the Illumina NovaSeq 6000 platform (Illumina, Inc., San Diego, CA, USA) using 2 × 150 bp paired-end chemistry, achieving an average coverage depth of approximately 100×. Primary and secondary analyses were conducted on the Illumina DRAGEN platform. The secondary analysis followed the GATK “best practices” pipeline, which includes Variant Quality Score Recalibration (VQSR). Sequence reads were aligned to the human reference genome (GRCh37/hg19) using the BWA algorithm.

The resulting variants were annotated and analyzed using multiple disease-related databases, including ClinVar, OMIM, GWAS Catalog, HGMD, and SwissVar. Common variants were filtered out based on allele frequency data from 1000 Genomes Phase 3, ExAC, EVS, dbSNP147, and an internal population database. The pathogenic potential of non-synonymous variants was evaluated using several in silico prediction algorithms: PolyPhen-2, SIFT, MutationTaster2, MutationAssessor, and LRT. Synonymous (silent) variants that do not result in an amino acid change within coding regions were excluded from the final report. Variant classification was performed according to the ACMG/AMP guidelines.

### 4.4. Statistical Analysis

All data were analyzed using MedCalc Statistical Software version 23.0.2 (MedCalc Software Ltd., Ostend, Belgium; https://www.medcalc.org; 2025). Descriptive statistics were employed to summarize the distribution of the variables. The Shapiro–Wilk test was used to assess the normality of the distribution. Based on distribution characteristics, appropriate parametric or non-parametric statistical tests were applied. In analyses involving repeated measures within the same group, the paired *t*-test (parametric) or the Wilcoxon signed-rank test (non-parametric) was employed. The significance level of *p* < 0.05 was considered statistically significant.

### 4.5. Unsupervised Machine Learning

During data analysis, a patented digital platform (German Patent Office, No. DE 20 2025 101 197 U1) was utilized to categorize study participants according to specific genetic findings obtained through whole-genome sequencing (WGS) [30]. The digital platform is a patented, machine learning-based software designed for patient categorization, or clustering, according to genetic variants in specific genes related to the metabolism of different nutrients. Through an unsupervised machine learning pipeline that integrates several key algorithms, the system identifies nutrigenetic clusters of patients sharing similar genetic profiles.

The first step in data analysis involved uploading the patient’s nutrigenetic report. The platform extracted the relevant data from the file using the file template, which is then forwarded to the clustering segment. The categorization process was done in two steps: dimensionality reduction and clustering. Due to the high complexity of the data in the nutrigenetic report, the features of each sample were compressed into two continuous components, using dimensionality reduction algorithms including principal component analysis (PCA) and uniform manifold approximation and projection (UMAP). When scaled to two dimensions, the clustering of data points was conducted using a Euclidean distance-based clustering algorithm, including K-Means. Using this data processing pipeline, the digital platform categorized patients into one of five nutrigenetic clusters. Feature importance for each cluster was inferred from topic-specific term probabilities derived from an unsupervised Latent Dirichlet Allocation (LDA) model, representing relative relevance within the learned topic structure.

**Figure 6:**
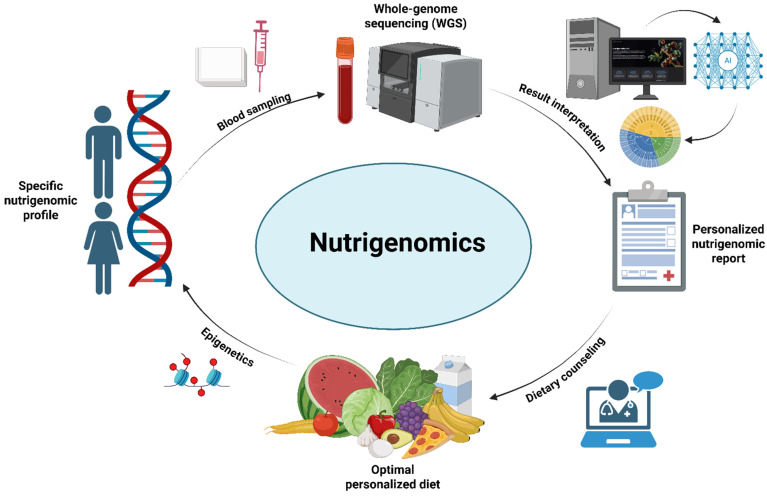
Integrative workflow for nutrigenomic analysis and personalized nutrition. Schematic representation of the nutrigenomic workflow integrating genomic data with personalized nutrition. Sequencing data are processed and interpreted using artificial intelligence (AI)-driven analytical tools to identify genetic variants relevant to nutrient metabolism. These results are summarized in a personalized nutrigenomic report, which guides dietary counseling and the formulation of an optimal personalized diet. The implemented dietary interventions can induce epigenetic modifications, contributing to an individualized nutrigenomic profile and establishing a feedback loop between genetic predisposition, nutrition, and gene expression (created with BioRender.com).

The second step of the analysis involved the generation of a personalized report for dietary recommendations. The internal database of the platform associates each of the five clusters with a set of foodstuffs that would be best suited for an individual with the established nutrigenetic profile. Once the patient has been sorted into a cluster, the nutrigenetic report is generated and can be exported for clinical use. Finally, the physician or nutritionist may use this report during consultation with the individual to more easily create a personalized dietary plan that takes genetics into account (Figure 6).

### 4.6. Longitudinal Protocol Description

In our study, all participants were given personalized nutrigenetic reports with recommended dietary measures. They were also instructed about the importance of physical activity for weight loss and encouraged to follow the advice given for the following 6 months. During that time, participants received frequent consultations from nutritionists. After 6 months, a second clinical assessment was made. The participants’ weight was measured, and again laboratory evaluation was identical to the first one.

## Figures and Tables

**Table 1:** Baseline clinical characteristics.

Variable	Mean ± SD	Median (IQR)	Min–Max
Age (y)	32 ± 9.62	27.5 (26–38.5)	21–50
Height (cm)	177 ± 11.62	175 (168.25–187.25)	161–195
Weight (kg)	163.54 ± 26.02	166.65 (146.78–177.93)	117–209.9
BMI (kg/m^2^)	52.09 ± 7.41	50.65 (47.98–56.63)	41.3–68.9

SD—standard deviation, IQR—interquartile range.

**Table 2:** Baseline biochemical characteristics.

Variable	Mean ± SD	Median (IQR)	Min–Max	Reference
Erythrocytes (×10^12^/L)	5.27 ± 0.44	5.2 (5.09–5.43)	4.6–6.1	4.34–5.72
Hemoglobin (g/L)	145.36 ± 17.08	148.5 (136.25–158.5)	108–166	138–175
Hematocrit (L/L)	0.45 ± 0.05	0.45 (0.42–0.48)	0.365–0.511	0.356–0.470
Platelets (×10^9^/L)	277.78 ± 77.83	267 (204.25–335)	186–421	158–424
Leukocytes (×10^9^/L)	8.65 ± 2.19	8.65 (7.37–9.2)	4.2–12.7	3.4–9.7
SE (mm/3.6 ks)	16.21 ± 10.13	14 (7.75–23.5)	4–35	4–24
CRP (mg/L)	10.2 ± 9.0	9.8 (4.3–12.2)	1.7–37.9	<5
Glucose (mmol/L)	6.08 ± 0.91	6 (5.27–6.55)	5.1–7.8	3.9–5.5
HbA1c (%)	5.49 ± 0.63	5.35 (5.1–5.7)	4.9–7.2	4.4–6.4
Insulin (mU/L)	141.7 ± 47.3	93.5 (93.5–181.6)	83.8–207.8	13.9–152.9
HOMA-IR (index)	2.7 ± 0.85	2.75 (1.9–3.38)	1.7–3.8	<2
Cholesterol (mmol/L)	5.52 ± 0.92	5.5 (4.87–6.17)	4.1–6.9	<5.0
HDL cholesterol (mmol/L)	1.07± 0.23	1 (0.9–1.2)	0.8–1.5	>1.2
LDL cholesterol (mmol/L)	3.91 ± 0.86	3.95 (3.32–4.62)	2.1–5.2	<1.4–3.0 *
Triglycerides (mmol/L)	1.93 ± 1.13	1.6 (1.23–1.8)	0.9–4.9	<1.7
Vitamin D (nmol/L)	47.36 ± 13.32	49 (35–54.75)	29–72	>75
Creatine kinase (U/L)	233.86 ± 193.15	180 (106–246.25)	89–713	<153
Uric acid (μmol/L)	417.86 ± 92.08	410 (370–460)	240–610	134–337

* Depending on risk factors; SD—standard deviation, IQR—interquartile range.

**Table 3:** Age and GlycanAge index distribution.

Variable	Mean ± SD	Median (IQR)	Min–Max
Age (y) *	32 ± 9.62	27.5 (26–38.5)	21–50
GlycanAge index *	56 ± 12.45	56 (49.25–62.5)	34–80

* Shapiro–Wilk test, *p* < 0.01; SD—standard deviation, IQR—interquartile range.

**Table 4:** Comparison of weight and BMI before and after 6 months of personalized dietary measures.

Variable	Mean ± SD	Median (IQR)	Min–Max
Weight (kg) *	163.54 ± 26.02	166.65 (146.78–177.93)	117–209.9
Weight after 6 months (kg)	108.32 ± 24.12	112.55 (88.45–125.95)	70.3–142.2
BMI (kg/m^2^) *	52.09 ± 7.41	50.65 (47.98–56.63)	41.3–68.9
BMI after 6 months (kg/m^2^)	34.6 ± 9.06	33.2 (29.18–38)	20.5–52.2

* Shapiro–Wilk test, *p* < 0.01; SD—standard deviation, IQR—interquartile range.

**Table 5:** Comparison of GlycanAge index before and after 6 months of personalized dietary measures.

Variable	Mean ± SD	Median (IQR)	Min–Max
GlycanAge index *	56 ± 12.45	56 (49.25–62.5)	34–80
GlycanAge index after 6 months	48 ± 14.83	45.5 (38.75–52.5)	31–80

* Shapiro–Wilk test, *p* < 0.01; SD—standard deviation, IQR—interquartile range.

## Data Availability

The original contributions presented in this study are included in the article. Further inquiries can be directed to the corresponding author.
